# Pharmacodynamics of Isavuconazole in a Rabbit Model of Cryptococcal Meningoencephalitis

**DOI:** 10.1128/AAC.00546-19

**Published:** 2019-08-23

**Authors:** Laura L. Kovanda, Charles Giamberardino, Laura McEntee, Dena L. Toffaletti, Kelly S. Franke, Andrew Bartuska, Gordon Smilnak, George C. de Castro, Katelyn Ripple, William W. Hope, John R. Perfect

**Affiliations:** a Astellas Pharma Global Development, Inc., Northbrook, Illinois, USA; b Duke University Medical Center, Durham, North Carolina, USA; c Antimicrobial Pharmacodynamics and Therapeutics, Department of Molecular and Clinical Pharmacology, Institute of Translational Medicine, University of Liverpool, Liverpool Health Partners, Liverpool, United Kingdom; d Royal Liverpool and Broadgreen University Hospital Trust, Liverpool Health Partners, Liverpool, United Kingdom

**Keywords:** *Cryptococcus neoformans*, cryptococcosis, fluconazole, isavuconazole, isavuconazonium sulfate, meningoencephalitis

## Abstract

*Cryptococcus* spp., important fungal pathogens, are the leading cause of fungus-related mortality in human immunodeficiency virus-infected patients, and new therapeutic options are desperately needed. Isavuconazonium sulfate, a newer triazole antifungal agent, was studied to characterize the exposure-response relationship in a rabbit model of cryptococcal meningoencephalitis. Rabbits treated with isavuconazonium sulfate were compared with those treated with fluconazole and untreated controls. The fungal burden in the cerebrospinal fluid was measured serially over time, while the yeast concentrations in the brain and the eye (aqueous humor) were determined at the end of therapy. The exposure impact of isavuconazonium sulfate dosing in the rabbit was linked using mathematical modeling. Similar significant reductions in the fungal burden in the brain and cerebrospinal fluid in rabbits treated with isavuconazonium sulfate and fluconazole compared with that in the untreated controls were observed. No dose-dependent response was demonstrated with isavuconazonium sulfate treatment in this study. The treatment of cryptococcal meningoencephalitis with isavuconazonium sulfate was similar to that with fluconazole. Dose-dependent reductions in yeast over time were not demonstrated, which limited our ability to estimate the pharmacodynamic target. Further nonclinical and clinical studies are needed in order to characterize the extent of the exposure-response relationship in cryptococcal meningoencephalitis. However, this study suggests that isavuconazonium sulfate, like fluconazole, could be beneficial in the setting of consolidation and maintenance therapy, rather than induction monotherapy, in high-burden cryptococcal meningoencephalitis.

## Abstract (precis)

*Cryptococcus* spp., important fungal pathogens, are the leading cause of fungus-related mortality in human immunodeficiency virus-infected patients, and new therapeutic options are desperately needed. Isavuconazonium sulfate, a newer triazole antifungal agent, was studied to characterize the exposure-response relationship in a rabbit model of cryptococcal meningoencephalitis. Rabbits treated with isavuconazonium sulfate were compared with those treated with fluconazole and untreated controls.

## TEXT

Infections caused by Cryptococcus neoformans and C. gattii are associated with excessive morbidity and mortality in patients with and without HIV infection. C. neoformans is the leading cause of fungus-related mortality in HIV-infected patients, especially in sub-Saharan Africa, where the HIV/AIDS epidemic is persistent (1). At the peak of the HIV epidemic, approximately 1 million cases of cryptococcosis were reported annually in patients with AIDS (1). In the developed world, cases of cryptococcosis declined over the same time period as the use of antiretroviral therapy increased. In contrast, the prevalence remained high in low- to middle-income countries (2). In the United States, it is estimated that nearly 3,400 hospitalizations occur yearly due to cryptococcal meningitis (3). Direct hospitalization-associated costs for cryptococcal meningitis amounted to approximately $54 million in 2009 (3).

Treatment of cryptococcal meningitis typically includes induction therapy with amphotericin B deoxycholate (0.7 to 1.0 mg/kg of body weight per day) or liposomal amphotericin B (AmBisome; 3 to 4 mg/kg per day intravenously) plus flucytosine (100 mg/kg per day orally) in four divided dosages for 1 week, followed by consolidation therapy with fluconazole (400 mg [6 mg/kg] per day orally) for a minimum of 8 weeks (4). In some areas of the world, fluconazole may be the only available agent for induction therapy (4). A high baseline cerebrospinal fluid (CSF) fungal burden, altered mental status, older age, and high peripheral white blood cell counts predict acute 2-week mortality. Furthermore, improved outcomes were associated with amphotericin-based treatment and prompt immune reconstitution with antiretroviral therapy (5).

Cryptococcal infection generally begins in the lungs, although meningitis is the most frequently encountered clinical manifestation of cryptococcosis among those with advanced immunosuppression. The disease is more properly characterized as meningoencephalitis rather than meningitis since the brain parenchyma is almost invariably involved on histologic examination (6). This understanding is critical when determining a successful treatment strategy, as central nervous system (CNS) penetration into the brain tissue as well as into the CSF is vital to improve the outcome.

Isavuconazonium sulfate, the water-soluble prodrug of the broad-spectrum triazole antifungal agent isavuconazole, is approved by the U.S. FDA for the treatment of invasive aspergillosis and invasive mucormycosis and by the European Medicines Agency for the treatment of invasive aspergillosis and invasive mucormycosis in patients for whom amphotericin B is inappropriate (7–9). Isavuconazole MIC values against *Cryptococcus* spp. ranged from 0.008 mg/liter to 0.5 mg/liter, with an overall modal MIC of 0.03 mg/liter, in a collection of more than 800 Cryptococcus neoformans isolates (10).

To further support an understanding of the effectiveness of isavuconazonium sulfate against cryptococcal infections, particularly in the CNS, we characterized the exposure-response relationship of isavuconazonium sulfate in a well-established rabbit model of cryptococcal meningoencephalitis caused by Cryptococcus neoformans.

## RESULTS

### Cryptococcus neoformans (H99) MIC values.

The geometric mean isavuconazole MIC value for the H99 strain of Cryptococcus neoformans was 0.008 mg/liter for isavuconazole and 1 mg/liter for fluconazole. Yeast grown from treatment day 8 of the isavuconazonium sulfate treatment animals showed similar MIC values.

### Animal model.

**FIG 1:**
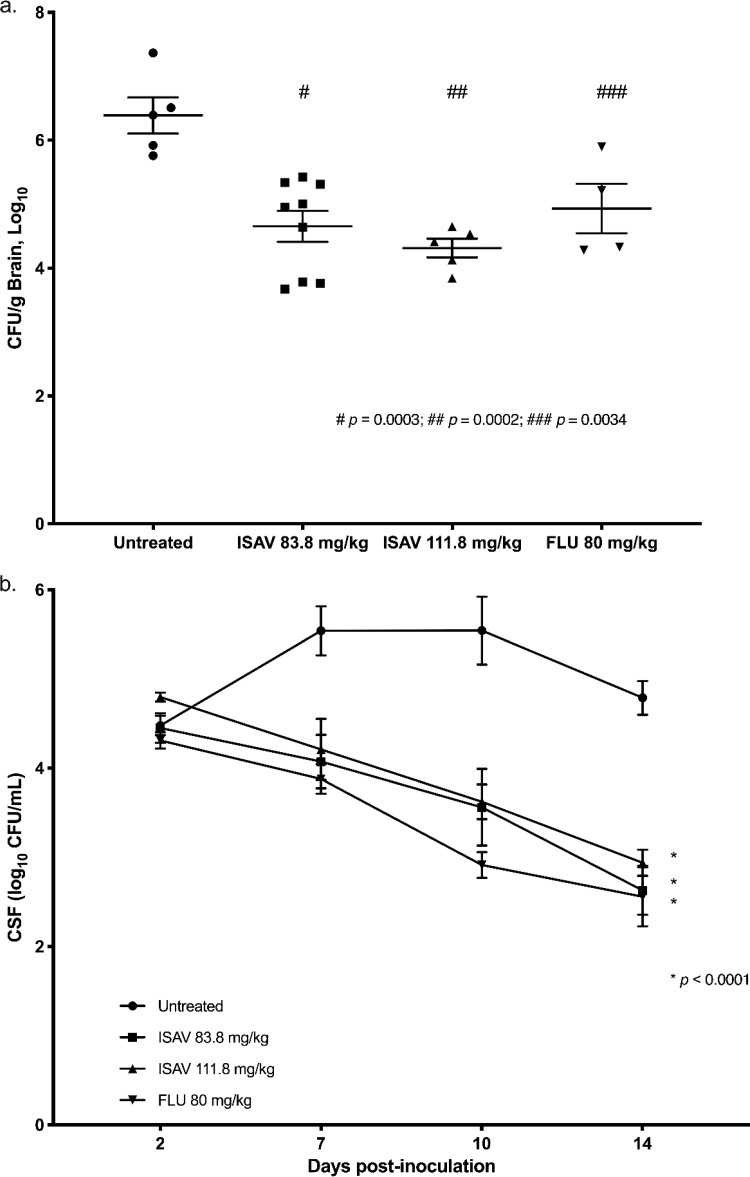
Changes in fungal burden in the rabbit brain (a) and CSF (b). (a) Fungal burden in brain. All treatment groups (isavuconazonium sulfate at 83.8 mg/kg and 111.8 mg/kg and fluconazole at 80 mg/kg) resulted in significant reductions in the number of log_10_ CFU per gram in the brain at the end of treatment compared with that in the brains of untreated rabbits (*P = *0.0003, *P = *0.0002, and *P = *0.0034, respectively, one-way ANOVA, Holm-Šidák’s multiple-comparison test). There was no statistically significant difference between either isavuconazonium sulfate treatment group versus the fluconazole treatment group (*P > *0.05). (b) Fungal burden in CSF. Significant changes in the number of log_10_ CFU per milliliter over time in CSF were demonstrated for all treatment groups (isavuconazonium sulfate at 83.8 mg/kg and 111.8 mg/kg and fluconazole at 80 mg/kg) versus that in the CSF of untreated rabbits (*P < *0.0001, Tukey’s multiple-comparison test). Note that not all animals survived to day 14. CSF, cerebrospinal fluid; ISAV, isavuconazonium sulfate; FLU, fluconazole.

Significant reductions in fungal burden were seen in the brain following administration of isavuconazonium sulfate doses of 83 mg/kg (equivalent to 45 mg/kg of isavuconazole) and 111.8 mg/kg (equivalent to 60 mg/kg of isavuconazole). These were comparable to 80 mg/kg of fluconazole. Both isavuconazonium sulfate and fluconazole treatments resulted in significant decreases in the fungal burden in the brain compared with that for the untreated controls (*P = *0.0003, *P = *0.0002, and *P = *0.0034, respectively, one-way analysis of variance [ANOVA], Holm-Šidák's multiple-comparison test) (Fig. 1a). Treatment with isavuconazonium sulfate and fluconazole resulted in significant reductions in the CSF fungal burden of yeast over time compared with that for the controls (*P < *0.0001, two-way ANOVA, Tukey’s multiple-comparison test) (Fig. 1b). Investigation of the residual fungal burdens in the eye (aqueous humor) showed that they were extremely variable and were not considered reliable; therefore, the results are not reported here.

### Pharmacokinetics.

Mean isavuconazole concentrations in the brain tissue (cerebrum, cerebellum, meninges) at the end of the experiment were similar between the isavuconazonium sulfate dose groups (Table 1). The mean ratio of brain-to-plasma isavuconazole concentrations in rabbits was 0.69 and 0.42 for the 83.8-mg/kg and 111.8-mg/kg doses, respectively. As with the brain tissue, the mean isavuconazole concentrations in the CSF did not increase with increasing dose (Table 1). The mean ratio of the CSF-to-plasma concentrations was 0.044 and 0.019 for the 83.8-mg/kg and 111.8-mg/kg doses, respectively (Table 1).

**TABLE 1:** Isavuconazole concentrations observed in CNS by dose

Parameter	Mean ± SD value at the following dose:	
	83.8 mg/kg	111.8 mg/kg
Brain isavuconazole concn (mg/liter)	1.15 ± 1.5	1.31 ± 0.96
CSF isavuconazole concn (mg/liter)	0.08 ± 0.049	0.05 ± 0.028
Ratio of brain-to-plasma concn	0.69 ± 0.69	0.42 ± 0.27
Ratio of CSF-to-plasma concn	0.044 ± 0.044	0.019 ± 0.006

### Pharmacokinetic/pharmacodynamic modeling.

Pharmacokinetic/pharmacodynamic modeling using the brain isavuconazole concentration data and fungal burden in the CNS was not possible because of the lack of temporal data for measures. Therefore, the pharmacokinetic/pharmacodynamic model focused on a link between plasma and CSF isavuconazole concentrations and the CSF fungal burden over time.

**FIG 2:**
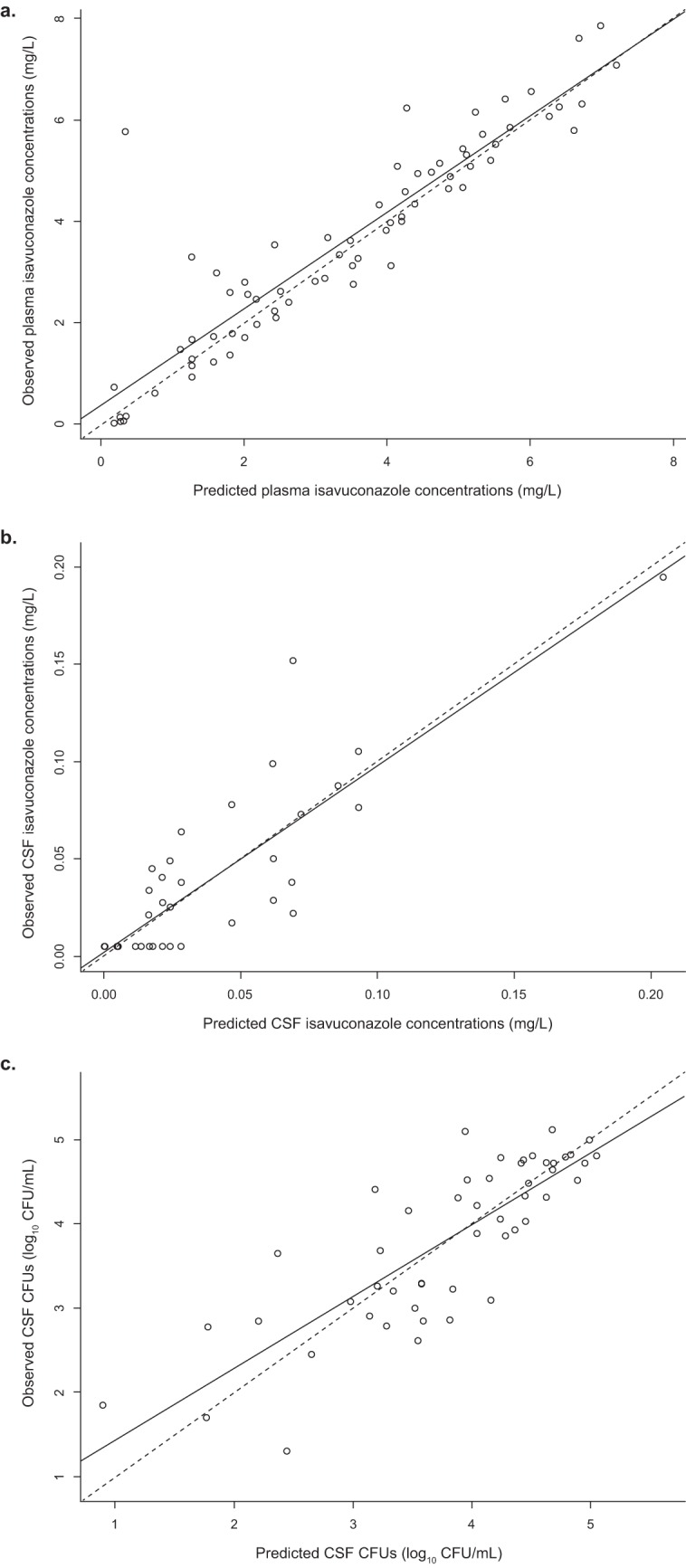
(a) Observed versus median posterior predicted isavuconazole plasma concentrations (in milligrams per liter) from the final model after the Bayesian step (*r*^2^ = 0.841, slope = 0.951 [95% confidence interval = 0.853 to 1.050], intercept = 0.366 [95% CI = −0.018 to 0.751]). (b) Observed versus median posterior predicted isavuconazole CSF concentrations (in milligrams per liter) from the final model after the Bayesian step (*r*^2^ = 0.745, slope = 0.958 [95% confidence interval = 0.79 to 1.13], intercept = 0.00198 [95% confidence interval = −0.00617 to 0.01010]). (c) Observed versus median posterior predicted number of log_10_ CFU per milliliter of CSF from the final model after the Bayesian step (*r*^2^ = 0.692, slope = 0.853 [95% CI = 0.69 to 1.02], intercept = 0.577 [95% confidence interval = −0.0697 to 1.22]). The dashed lines are the line of unity, where the observed concentration equals the predicted concentration. CSF, cerebrospinal fluid.

The fit of the model to the plasma and CSF isavuconazole concentration data and the number of yeast CFU in the CSF over time was acceptable, based on visual inspection of the median observed-versus-predicted plots. A linear regression had a coefficient of determination (*r*^2^) of 0.841 (slope = 0.951), 0.745 (slope = 0.958), and 0.692 (slope = 0.853) after the Bayesian step for the isavuconazole concentrations in the plasma and CSF and the CSF fungal burden, respectively (Fig. 2a, b, and c). The estimates of bias and imprecision were also acceptable (for the concentration in plasma, −0.122 and 1.19, respectively; for the concentration in CSF, −0.0246 and 2.02, respectively; for the number of CFU in CSF, 0.00863 and 0.0207, respectively). The observed-versus-predicted plots using the mean parameter values were similar (data not shown). The mean parameter estimates are included in Table 2.

**TABLE 2:** Values for each parameter estimated from the rabbit population pharmacokinetic/pharmacodynamic-linked model

Parameter^*a*^ (units)	Mean	SD
*K_a_* (h^−1^)	3.196	4.62
CL/*F* (liters/h)	2.639	2.26
*Vc*/*F* (liters)	12.255	5.79
*Vb*/*F* (liters)	165.876	51.75
*Kcp* (h^−1^)	17.942	9.82
*Kpc* (h^−1^)	20.135	9.83
*Kcm* (h^−1^)	13.959	13.52
*Kmc* (h^−1^)	16.593	9.00
*Kg*max (log_10_ CFU/ml)	0.027	0.02
*Hg*	13.670	9.13
*C*50*g* (mg/liter)	1.754	1.61
IC (no. of log_10_ CFU/ml)	28,870.429	19,162.86
*kk*max (no. of log_10_ CFU/ml)	11.168	5.19
popmax (no. of log_10_ CFU/ml)	409,294.206	449,097.00
*Hk*	3.697	2.66
*C*50*k* (mg/liter)	1.928	1.42

a *Vc*/*F*, volume of distribution of the central compartment; *Vb*/*F*, volume of distribution of the brain compartment; IC, initial condition. The other abbreviations are defined in the text.

The median Bayesian posterior pharmacokinetic parameters from the pharmacokinetic/pharmacodynamic model were used to estimate the area under the concentration-time curve (AUC) from 0 h to 24 h (AUC_0–24_) for plasma and CSF, as well as the area under the curve for the CSF fungal burden-versus-time relationship (Table 3). Although there was a nearly 2-fold increase in the plasma isavuconazole exposures, drug exposure in the CSF did not increase. Not surprisingly, this lack of increase in exposure by dose in the CSF compartment resulted in minimal differences in response by dose (Table 3). This lack of change in exposure and effect by dose resulted in the inability to explore and characterize the pharmacodynamic target (the 50% effective concentration) for the response in the inhibitory sigmoid maximum-effect model with this data set.

**TABLE 3:** Mean plasma and CSF AUC_0–24_ for each dose group and mean area under the curve for number of log_10_ CFU per milliliter over time for *Cryptococcus* spp. in CSF

Parameter	Value at the following dose:	
	83.8 mg/kg	111.8 mg/kg
Plasma AUC_0–24_ (mg · h/liter)	50.83 ± 33.302	99.83 ± 31.292
CSF AUC_0–24_ (mg · h/liter)	2.87 ± 2.013	1.35 ± 0.598
No. of log_10_ CFU/ml vs time	1,304.36 ± 240.36	1,379.01 ± 273.89
Decline in no. of log_10_ CFU/ml	1.78 ± 0.69	1.89 ± 0.33

## DISCUSSION

Treatment options for cryptococcal infections are limited, and the available antifungal agents have not changed substantially in the past ∼20 years. It was not until recently that characterization of the exposure-response relationship with animal models and bridging studies became available to guide dosing in clinical studies (11, 12). These efforts led to modifications in the sequence and duration of induction therapy, as well as changes to the dosage of the three recommended agents: amphotericin B, flucytosine, and fluconazole (4). Access to all three medications in countries with limited resources—where the incidence of the infection is the highest—is inconsistent (2). Fluconazole is the only agent available in some of these countries. In the current study, isavuconazonium sulfate demonstrated significant reductions in the CSF and brain fungal burden in rabbits infected with Cryptococcus neoformans (H99) compared with that in untreated rabbits and reductions similar to those achieved with fluconazole. However, a dose-dependent response was not demonstrated for isavuconazole for the dosages used in this study.

The isavuconazonium sulfate regimens used to treat invasive mold infections result in total drug plasma AUCs of approximately 90 mg · h/liter (13). There are limited data on CNS penetration. One report that detailed the response of patients receiving isavuconazonium sulfate dosages ranging from 372 mg/day (equivalent to 200 mg/day isavuconazole) to 1,116 mg/day (equivalent to 600 mg/day isavuconazole) is available (14). The CSF concentrations measured during treatment are summarized in Table 4. The ratio of the CSF-to-plasma concentration was consistently 0.008:0.011 and did not increase with increasing dose. If one assumes similar CSF penetration in rabbits and humans, the CSF concentrations achieved with doses above 372 mg/day are closer to those concentrations observed in the rabbits. Limited data on the human brain concentrations of isavuconazole are available, but a brain-to-plasma concentration ratio of 0.9 has been reported for one patient (15).

**TABLE 4:** Plasma and CSF concentrations from two patients treated with isavuconazonium sulfate^*b*^

Patient and dose (mg q24h)	Concn (mg/liter) in:		Ratio of CSF to plasma concn
	Serum	CSF	
Patient 1			
372	3.608	0.0296^*a*^	0.008
1,116	11.936	0.0917^*a*^	0.008
1,116	16.389	0.1312	0.008
744	13.924	0.0976^*a*^	0.007
558	11.749	0.109	0.009
558	9.371	NA	NA
372	6.227	NA	NA
Patient 2			
372	4.489	0.0228^*a*^	0.005
372	3.798	0.0405^*a*^	0.011

a Values of <0.1 mg/liter are extrapolated and are not actual measured drug concentrations.

b Adapted from reference 14 with permission. NA, not available; q24h, every 24 h.

Given that a significant proportion of the clinical impact of cryptococcal disease in humans relates to involvement of the brain, the extent of penetration into the cerebral parenchyma is important. Data from a study of radiolabeled isavuconazonium sulfate administered to rats showed a brain-to-plasma concentration ratio of approximately 1.8:1 (16). A similar ratio has been reported from infected mice after receiving isavuconazonium sulfate for 14 days (17). An *in vivo* model of CNS disease has been conducted with isavuconazonium sulfate in mice infected with two strains of Cryptococcus neoformans (USC 1597 and H99) (17). Significant improvements in survival and reductions in the fungal burden in the cerebrum were observed with isavuconazonium sulfate treatment compared with the results for the controls, and the effects were similar to those of treatment with fluconazole.

In the clinical setting, nine patients diagnosed with cryptococcosis were treated with isavuconazonium sulfate in an open-label study (the VITAL study) (17). Eight patients were alive through ≥84 days, and six were considered to be treatment successes.

As some yeasts persisted in the subarachnoid space for 12 days after treatment, we checked several colonies from the rabbit CSF after 8 days of treatment, and there was no change in their original MICs, suggesting no evidence of direct selection for drug-resistant isolates (data not shown). The yeasts showed a nonsignificant reduction in the eyes following treatment with both isavuconazonium sulfate and fluconazole compared with the levels in the untreated controls.

The current study has several limitations. First, there were insufficient data available to understand the temporal drug exposure in the cerebrum and eyes, eliminating an ability to characterize the exposure-response relationships in these tissues. Only a single yeast strain was studied in the rabbit model, which limits an ability to understand the potential impact of pharmacodynamic variability on the clinical utility of isavuconazonium sulfate for cryptococcal meningitis. Despite these limitations, treatment with isavuconazonium sulfate and fluconazole provided similar significant reductions in the cryptococcal burden in both the brain and CSF. Additional preclinical and clinical studies are required to further define the clinical utility and potential indications of isavuconazonium sulfate for cryptococcal meningoencephalitis.

## MATERIALS AND METHODS

### Organism.

A clinical strain of Cryptococcus neoformans (H99) was used. H99 yeasts were stored at −80°C in 25% glycerol. The isavuconazole and fluconazole MICs were tested using the CLSI M27-A3 macrodilution methodology. Briefly, RPMI 1640 broth inoculated with 0.5 × 10^3^ to 2.5 × 10^3^ CFU/ml was incubated for 72 h at 35°C. The endpoint for determination of the MIC was defined as a ≤50% reduction in growth relative to the growth of control yeasts without drug exposure.

### Rabbit model of cryptococcal meningitis.

Male New Zealand White rabbits weighing 2 to 3 kg were used; this model has been described previously (12). The Duke University IACUC approved the protocol prior to study initiation.

Rabbits were individually housed and maintained with water and standard rabbit feed *ad libitum*. Immunosuppression was induced using hydrocortisone acetate at 5 mg/kg by intramuscular injection daily starting on day −1 and continuing through day 13 postinfection. The rabbits were given hand-fed vegetables and supplemental subcutaneous fluids throughout the study. They were weighed daily throughout the study. Rabbits were sedated with an intramuscular injection of a mixture of ketamine (30 mg/kg) and xylazine (3 mg/kg) prior to intracisternal inoculation and cisternal taps. Intravenous (i.v.) yohimbine was used to reverse the sedation, and rabbits were recovered under supplemental heat with continuous monitoring. Prior to euthanasia, the rabbits were sedated with ketamine and xylazine (40 mg/kg and 5 mg/kg, respectively).

### Pharmacokinetic blood collections.

Blood collection was performed on day 8 postinfection. For pharmacokinetic blood draws, rabbits were sedated with acepromazine at 1 mg/kg, and a catheter was placed in the ear artery and sutured in place. For each blood draw, the catheter was flushed with heparinized saline. A presample was then collected and discarded to remove any residual heparin, and then the sample was collected from the catheter. Blood samples were taken in EDTA plasma separator tubes at the indicated time points after drug administration. Plasma was isolated by centrifugation and stored at −80°C prior to testing.

### Pharmacokinetic tissue collections and processing.

Brain and meningeal tissue was collected at the end of the experiment. Tissue samples were weighed and transferred to a homogenization tube. The weight was multiplied by 3 for brain and 5 for meninges to adjust the volume of the reconstitution solvent to that of the samples. Tissues were homogenized for 30 s at 4 m/s, and homogenization was repeated as necessary.

### Inoculation.

C. neoformans strain H99 was grown at 30°C for 2 to 3 days on yeast extract-peptone-dextrose (YPD) plates. A single colony was selected, and a 25-ml YPD broth culture was initiated and grown for 2 days at 30°C in a shaker incubator. The organisms were washed twice in phosphate-buffered saline (PBS) and diluted to 3.9 × 10^6^ cells/ml in PBS. In previous studies, we used 1 × 10^8^ cells of C. neoformans per inoculation, but a pilot study indicated that a lower inoculum, 1 × 10^6^ cells, was required for a 14-day study. A volume of 0.3 ml of inoculum was loaded into 3-ml syringes with 25-gauge needles. The yeast inoculum was administered by injecting 0.3 ml intracisternally on day −2 before dosing. The rabbits were injected with yohimbine at 0.2 mg/kg i.v. As mentioned above, the rabbits were given supplemental subcutaneous fluids during recovery from sedation.

### Treatment.

Isavuconazole was administered as a water-soluble prodrug, isavuconazonium sulfate, via oral gavage. The drug was stored in sterilized glass vials at −20°C. Prior to dosing each day, an aliquot was removed and a volume of pH 4 USP-grade water was added to the aliquot to yield a solution of 60 mg/ml of prodrug. Prodrug doses included 83.8 mg/kg and 111.8 mg/kg once daily, which delivered 45 mg/kg and 60 mg/kg of isavuconazole, respectively, to the animal. Fluconazole was purchased as an oral suspension and administered i.v. at a dose of 80 mg/kg per day. It was reconstituted with USP-grade water to a final concentration of 40 mg/ml. Untreated controls received saline. Drug dosing began at 48 h postinoculation and continued for 12 consecutive days.

### Fungal burden of tissue.

All rabbits were humanly sacrificed after the final CSF collection, either on day 10 or at the time of sacrifice. Brains were removed and then dissected sagittally for quantitative cultures and drug level analysis. The fungal burden in brain (cut into three sections) and eyes (vitreous humor and aqueous humor) was measured. Tissue was placed in preweighed tubes containing 1 ml of PBS and then weighed again to find the net weight of the tissue. Tissue was homogenized using a Pro200 homogenizer (Pro Scientific, Oxford, CT, USA) in a biological safety cabinet. The tissue homogenates were 10-fold serially diluted and then plated on YPD with chloramphenicol agar. Cultures were grown for 3 days at 30°C. The numbers of CFU were quantified as the numbers of CFU per gram of tissue. The fungal burden within the CSF from the subarachnoid space was measured from serial samples taken at 48, 144, and 240 h postinoculation via an intracisternal tap with a 3-ml syringe and a 25-gauge needle, serially diluted (1:10) in PBS in 1 ml, plated on YPD with chloramphenicol agar, and incubated at 30°C for 2 to 3 days, and then the viable yeast colonies were counted. Calculations of the number of CFU per milliliter were as follows: (number of CFU counted × dilution)/0.1 ml. The remaining CSF was centrifuged, and the supernatant was stored at −80°C.

### Isavuconazole bioanalytical assay.

Isavuconazole concentrations in rabbit plasma, CSF, and brain were measured using a Thermo Fisher Scientific Vanquish (Waltham, MA, USA) ultraperformance liquid chromatograph coupled with a Thermo Fisher Scientific Q Exactive Focus (Waltham, MA, USA) mass spectrometer. The method used an ACE 5 C_18_-AR column (50 by 3.0 mm; supplier, Hichrom Ltd, Reading, UK) and a 3-μl injection volume. A standard curve encompassing 15 to 40,000 ng/ml was constructed from stock solutions of isavuconazole at 1 mg/ml in dimethyl sulfoxide. Chromatographic separation was achieved using gradient conditions starting with 35:65 (0.1% formic acid in water as mobile phase A and 0.1% formic acid in acetonitrile as mobile phase B). Mobile phase B was increased to 95% from 0.2 to 1.0 min and held for 1.6 min. At 1.7 min, mobile phase B was reduced to 65%. This was all at a flow rate of 0.6 ml/min. Isavuconazole was detected using the exact mass in positive ion mode (438.1195). Isavuconazole eluted after 1.0 min. The limit of detection was 15 ng/ml, and the assay was linear over the concentration range of 15 to 40,000 ng/ml. The quality control intra- and interday accuracy was 98.7 to 104.6%. The quality control coefficient of variation percentage was 1.1 to 8.4%.

### Pharmacokinetic/pharmacodynamic mathematical modeling.

Population pharmacokinetic/pharmacodynamic mathematical modeling was performed using nonparametric estimation with Pmetrics software (v.1.5.2; University of Southern California, Los Angeles, CA, USA) (18) and fitted to the rabbit plasma and CSF isavuconazole concentration data and the number of CSF CFU. Data were weighted by the inverse of the estimated assay variance. The linked pharmacokinetic/pharmacodynamic model was constructed using the following set of differential equations:

(1) $$\frac{\mathrm{dX}(1)}{\mathrm{dt}}=-Ka\cdot X(1)$$

(2) $$\frac{\mathrm{dX}(2)}{\mathrm{dt}}=Ka\cdot X(1)-\left[\left(\frac{\text{CL}}{V}\right)\cdot X(2)\right]-\mathrm{Kcp}\cdot X(2)+\mathrm{Kpc}\cdot X(4)-\mathrm{Kcm}\cdot X(2)+\mathrm{Kmc}\cdot X(3)$$

(3) $$\frac{\mathrm{dX}(3)}{\mathrm{dt}}=\mathrm{Kcm}\cdot X(2)+\mathrm{Kmc}\cdot X\left(3\right)$$

(4) $$\frac{\mathrm{dX}(4)}{\mathrm{dt}}=\mathrm{Kcp}\cdot X(2)+\mathrm{Kpc}\cdot X\left(4\right)$$

(5) $$\frac{\mathrm{dX}\text{(}5\text{)}}{\mathrm{dt}}\text{= }\mathrm{Kg}\text{max}\cdot \left(1-\left\{\frac{{\left[\frac{X(3)}{V}\right]}^{\mathrm{Hg}}}{C50g^{\mathrm{Hg}}+{\left[\frac{X(3)}{V}\right]}^{\mathrm{Hg}}}\right\}\right)\cdot X(5)\cdot \left\{1-\left[\frac{X(5)}{\text{popmax}}\right]\right\}-\mathrm{kk}\text{max}\cdot X(5)\left\{\frac{{\left[\frac{X(3)}{V}\right]}^{Hk}}{C50k^{\mathrm{Hk}}+{\left[\frac{X(3)}{V}\right]}^{Hk}}\right\}$$

The first four equations describe the pharmacokinetics of isavuconazole, (compartment 1, theoretical absorptive compartment for oral administration; compartment 2, central compartment; compartment 3, CSF compartment; and compartment 4, peripheral compartment). CL is the clearance and defined as the amount of drug being cleared from the central compartment over time, and *V* is the volume of the central compartment. *Ka* is the first-order absorption constant; *Kcp*, *Kpc*, *Kcm*, and *Kmc* are the rate constants describing the flow of drug to and from compartments 2, 3, and 4. Equation 5 describes the rate of change of the *Cryptococcus* yeasts as the number of log_10_ CFU per milliliter of CSF. *Kg*max represents the maximum rate of growth, *Hg* is the slope function for growth, *C*50*g* is the amount of drug where there is half-maximal growth, popmax is the theoretical maximum density of yeasts in the CSF, *kk*max is the maximum rate of growth inhibition, *C*50*k* is the amount of drug where there is half-maximal growth inhibition, *Hk* is the slope function for growth inhibition, *t* is time, and *X* is amount of drug.

The final model was assessed by a visual inspection of the observed-versus-predicted concentration values plotted over time after the Bayesian step, the coefficient of determination (*r*^2^) from the linear regression of the observed-versus-predicted values, and evaluation of the estimated bias (mean weighted error) and precision (adjusted mean weighted squared error). After fitting the model to the pharmacokinetic/pharmacodynamic data, the Bayesian posterior estimates for each rabbit were used to estimate the plasma and CSF concentration-time profiles for isavuconazole and the change in the number of yeast CFU over time in the CSF for each rabbit. CSF AUCs and the area under the curve for the number of log_10_ CFU per milliliter of *Cryptococcus* spp. in the CSF over time for each rabbit were calculated by integration from the simulated concentration-time profiles (on the last day of dosing for plasma) in Pmetrics.

Statistical comparisons were performed in GraphPad Prism software (version 8.0; GraphPad Software, San Diego, CA, USA).

## References

[1] Park BJ, Wannemuehler KA, Marston BJ, Govender N, Pappas PG, Chiller TM 2009 Estimation of the current global burden of cryptococcal meningitis among persons living with HIV/AIDS. AIDS 23:525–530. doi:10.1097/QAD.0b013e328322ffac. 19182676

[2] Rajasingham R, Smith RM, Park BJ, Jarvis JN, Govender NP, Chiller TN, Denning DW, Loyse A, Boulware DR 2017 Global burden of disease of HIV-associated cryptococcal meningitis: an updated analysis. Lancet Infect Dis 17:873–881. doi:10.1016/S1473-3099(17)30243-8. 28483415 PMC5818156

[3] Pyrgos V, Seitz AE, Steiner CA, Prevots DR, Williamson PR 2013 Epidemiology of cryptococcal meningitis in the US: 1997–2009. PLoS One 8:e56269. doi:10.1371/journal.pone.0056269. 23457543 PMC3574138

[4] World Health Organization. 2018 Guidelines for the diagnosis, prevention and management of cryptococcal disease in HIV-infected adults, adolescents and children: supplement to the 2016 consolidated guidelines on the use of antiretroviral drugs for treating and preventing HIV infection. WHO Guidelines Approved by the Guidelines Review Committee, World Health Organization, Geneva, Switzerland https://www.ncbi.nlm.nih.gov/books/NBK531449/. Accessed 25 June 2019. 30285342

[5] Jarvis JN, Bicanic T, Loyse A, Namarika D, Jackson A, Nussbaum JC, Longley N, Muzoora C, Phulusa J, Taseera K, Kanyembe C, Wilson D, Hosseinipour MC, Brouwer AE, Limmathurotsakul D, White N, van der Horst C, Wood R, Meintjes G, Bradley J, Jaffar S, Harrison T 2014 Determinants of mortality in a combined cohort of 501 patients with HIV-associated cryptococcal meningitis: implications for improving outcomes. Clin Infect Dis 58:736–745. doi:10.1093/cid/cit794. 24319084 PMC3922213

[6] Lee SC, Dickson DW, Casadevall A 1996 Pathology of cryptococcal meningoencephalitis: analysis of 27 patients with pathogenetic implications. Hum Pathol 27:839–847. doi:10.1016/S0046-8177(96)90459-1. 8760020

[7] Astellas Pharma US Inc. 2015 Cresemba (isavuconazonium sulfate) prescribing information. http://www.astellas.us/docs/cresemba.pdf. Accessed 8 March 2019.

[8] European Medicines Agency. 2015 Cresemba (isavuconazonium sulfate) product information. http://www.ema.europa.eu/ema/index.jsp?curl=pages/medicines/human/medicines/002734/human_med_001907.jsp&mid=WC0b01ac058001d124. Accessed 8 March 2019.

[9] Thompson GR III, Wiederhold NP 2010 Isavuconazole: a comprehensive review of spectrum of activity of a new triazole. Mycopathologia 170:291–313. doi:10.1007/s11046-010-9324-3. 20524153

[10] Espinel-Ingroff A, Chowdhary A, Gonzalez GM, Guinea J, Hagen F, Meis JF, Thompson GR III, Turnidge J 2015 Multicenter study of isavuconazole MIC distributions and epidemiological cutoff values for the *Cryptococcus neoformans*- *Cryptococcus gattii* species complex using the CLSI M27-A3 broth microdilution method. Antimicrob Agents Chemother 59:666–668. doi:10.1128/AAC.04055-14. 25313209 PMC4291414

[11] Sudan A, Livermore J, Howard SJ, Al-Nakeeb Z, Sharp A, Goodwin J, Gregson L, Warn PA, Felton TW, Perfect JR, Harrison TS, Hope WW 2013 Pharmacokinetics and pharmacodynamics of fluconazole for cryptococcal meningoencephalitis: implications for antifungal therapy and in vitro susceptibility breakpoints. Antimicrob Agents Chemother 57:2793–2800. doi:10.1128/AAC.00216-13. 23571544 PMC3716186

[12] Livermore J, Howard SJ, Sharp AD, Goodwin J, Gregson L, Felton T, Schwartz JA, Walker C, Moser B, Müller W, Harrison TS, Perfect JR, Hope WW 2014 Efficacy of an abbreviated induction regimen of amphotericin B deoxycholate for cryptococcal meningoencephalitis: 3 days of therapy is equivalent to 14 days. mBio 5:e00725-13. doi:10.1128/mBio.00725-13. 24473125 PMC3903272

[13] Kovanda LL, Desai AV, Lu Q, Townsend RW, Akhtar S, Bonate P, Hope WW 2016 Isavuconazole population pharmacokinetic analysis using non-parametric estimation in patients with invasive fungal disease (results from the VITAL study). Antimicrob Agents Chemother 60:4568–4576. doi:10.1128/AAC.00514-16. 27185799 PMC4958143

[14] Everson N, Smith J, Garner D 2015 Successful treatment of contaminated epidural steroid associated fungal meningitis with isavuconazole, abstr P0231 Abstr 25th Eur Cong Clin Microbiol Infect Dis, Copenhagen, Denmark European Society of Clinical Microbiology and Infectious Diseases, Basel, Switzerland https://www.escmid.org/escmid_publications/escmid_elibrary/. Accessed 25 June 2019.

[15] Schmitt-Hoffmann AH, Kato K, Townsend R, Potchoiba MJ, Hope WW, Andes D, Spickermann J, Schneidkraut MJ 2017 Tissue distribution and elimination of isavuconazole following single and repeat oral-dose administration of isavuconazonium sulfate to rats. Antimicrob Agents Chemother 61:e01292-17. doi:10.1128/AAC.01292-17. 28971866 PMC5700325

[16] Wiederhold NP, Kovanda L, Najvar LK, Bocanegra R, Olivo M, Kirkpatrick WR, Patterson TF 2016 Isavuconazole is effective for the treatment of experimental cryptococcal meningitis. Antimicrob Agents Chemother 60:5600–5603. doi:10.1128/AAC.00229-16. 27324761 PMC4997854

[17] Thompson GR III, Rendon A, Ribeiro Dos Santos R, Queiroz-Telles F, Ostrosky-Zeichner L, Azie N, Maher R, Lee M, Kovanda L, Engelhardt M, Vazquez JA, Cornely OA, Perfect JR 2016 Isavuconazole treatment of cryptococcosis and dimorphic mycoses. Clin Infect Dis 63:356–362. doi:10.1093/cid/ciw305. 27169478 PMC4946023

[18] Neely MN, van Guilder MG, Yamada WM, Schumitzky A, Jelliffe RW 2012 Accurate detection of outliers and subpopulations with Pmetrics, a nonparametric and parametric pharmacometric modeling and simulation package for R. Ther Drug Monit 34:467–476. doi:10.1097/FTD.0b013e31825c4ba6. 22722776 PMC3394880

